# Intranasal “painless” Human Nerve Growth Factors Slows Amyloid Neurodegeneration and Prevents Memory Deficits in App X PS1 Mice

**DOI:** 10.1371/journal.pone.0037555

**Published:** 2012-05-30

**Authors:** Simona Capsoni, Sara Marinelli, Marcello Ceci, Domenico Vignone, Gianluca Amato, Francesca Malerba, Francesca Paoletti, Giovanni Meli, Alessandro Viegi, Flaminia Pavone, Antonino Cattaneo

**Affiliations:** 1 European Brain Research Institute, Rome, Italy; 2 Scuola Normale Superiore, Pisa, Italy; 3 Institute of Neuroscience, Consiglio Nazionale delle Ricerche, Rome, Italy; Case Western Reserve University, United States of America

## Abstract

Nerve Growth Factor (NGF) is being considered as a therapeutic candidate for Alzheimer's disease (AD) treatment but the clinical application is hindered by its potent pro-nociceptive activity. Thus, to reduce systemic exposure that would induce pain, in recent clinical studies NGF was administered through an invasive intracerebral gene-therapy approach. Our group demonstrated the feasibility of a non-invasive intranasal delivery of NGF in a mouse model of neurodegeneration. NGF therapeutic window could be further increased if its nociceptive effects could be avoided altogether. In this study we exploit forms of NGF, mutated at residue R100, inspired by the human genetic disease HSAN V (Hereditary Sensory Autonomic Neuropathy Type V), which would allow increasing the dose of NGF without triggering pain. We show that “painless” hNGF displays full neurotrophic and anti-amyloidogenic activities in neuronal cultures, and a reduced nociceptive activity *in vivo*. When administered intranasally to APPxPS1 mice ( n = 8), hNGFP61S/R100E prevents the progress of neurodegeneration and of behavioral deficits. These results demonstrate the *in vivo* neuroprotective and anti-amyloidogenic properties of hNGFR100 mutants and provide a rational basis for the development of “painless” hNGF variants as a new generation of therapeutics for neurodegenerative diseases.

## Introduction

Nerve Growth Factor (NGF) [1] has a considerable therapeutic potential for Alzheimer's disease (AD), not merely as a long-lasting cholinergic maintenance [2], [3], [4] and neuroprotective agent [5], [6], but also as a direct anti-amyloidogenic factor [7], [8]. However, the development of an NGF-based therapy for neurodegenerative diseases remains a considerable challenge [9], due to limited access of NGF to the brain [10] and to its potent nociceptive actions in animals and humans [11], [12].

Current strategies for NGF therapy in AD use highly invasive approaches, such as a neurosurgical intracerebroventricular injection of NGF [13] or a parenchimal injection of cells secreting hNGF (human NGF) [14] or of viruses harboring hNGF gene [15].

To fully exploit the therapeutic potential of NGF in a noninvasive manner, its therapeutic window must be improved, by increasing the brain distribution, while limiting NGF pain-inducing actions [7]. The intranasal delivery [16] represents a viable option to non invasively increase NGF biodistribution in the brain [17], where it exerts anti-neurodegenerative actions [18], [19], [20]. NGF intranasal delivery minimizes the build-up of peripheral NGF concentration, even if residual leakage and absorption of NGF into the blood stream, from the nasal compartment, has been shown [16], [17].

NGF therapeutic window could be further increased if its nociceptive effects could be avoided altogether. To this aim, we described a “painless” form of NGF [21], linked to the rare human genetic disease HSAN V (Hereditary Sensory Autonomic Neuropathy Type V) [22]. In HSAN V patients, a mutation in the NGFB gene ( exon 3, nt C661T ), changing arginine R100 in mature NGF to a tryptophan [22], determines the complete loss of pain perception without affecting most neurological functions [23]

The fact that the NGF mutation R100W appears to separate the effects of NGF on CNS development from those involved in the activation of peripheral pain pathways, provides a basis for designing “painless” NGF variant molecules [7], [21], [24]. In particular, we demonstrated that the hNGFR100E mutant displays a full neurotrophic activity in cultured neurons, while showing a reduced nociceptive activity *in vivo* [21], via a selective alteration of TrkA versus p75NTR binding and signaling [21], [24].

In this paper, we characterize a double mutant of human NGF (hNGFP61S/R100E; Accession number: SPIN000006237) that, in addition to the pain-related R100E mutation, harbors a second “tagging” P61S mutation [25]. The anti-neurodegenerative properties of hNGFP61S/R100E protein were assessed in a disease-relevant primary neuron model, as well as in two animal models for AD, based on different mechanisms. In particular, this study addresses the question whether the intranasal administration of NGF-based molecules to Familial Alzheimer Disease-based mouse models might exert neuroprotection and prevent the progression of the neuropathological and behavioral deficits.

## Results

### Design and receptor binding properties of hNGFP61S/R100E protein

The therapeutic window for NGF in AD would be improved by the availability of NGF variants that, while retaining neurotrophic activity, would display a reduced nociceptive activity in vivo [7], [21].

**Figure 1:**
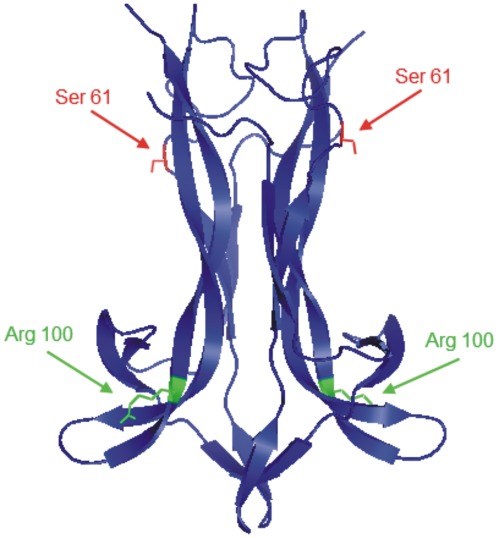
Structural localization of the residues Ser 61 and Arg 100 on mouse NGF (PDB: 1BET). The NGF dimer is depicted in blue. The Ser 61 residues on the two monomers are highlighted in red and the Arg 100 in green. The cartoon representation was created with Pymol (http://www.pymol.org). The scaffold of mouse NGF was used for this figure for illustration purposes, because the loop containing Pro 61 in the human NGF is missing in the available PDB structures. The mutant human NGF protein used in this study (hNGFP61S/R100E) is based, instead, on human NGF, in which residue Pro61 is substituted by its mouse counterpart Ser61, and residue Arg100 is substituted by Glu100.

To test whether the administration of recombinant forms of NGF to APP-based mouse models might exert a neuroprotective effect and slow the progression of neurodegeneration, we exploited the double mutant hNGFP61S/R100E (Figure 1).

The design of this mutant was based on the single nucleotide missense mutation in the NGFB gene, found in Hereditary Sensory and Autonomic Neuropathy type V (HSAN V) patients [22], that changes residue R100, in mature hNGF, into a W residue. We recently showed that the R100 mutation does not affect hNGF binding affinity for TrkA, while it reduces binding affinity for p75NTR by two orders of magnitude [24]. It was also found that R100W hNGF mutants have very similar receptor binding and signaling properties to an engineered R100E mutant [21], [24], with the latter giving much higher yields, when expressed and purified from E. coli cells. For this reason, we used the R100E hNGF mutants for in vivo studies, demonstrating that hNGFR100E has a nociceptive activity which is much weaker than that of wild type hNGF [21].

For the present study, the R100E mutation was inserted in the context of a recombinant form of human NGF “tagged” with a single residue epitope (hNGFP61S), which replaces the Pro residue at position 61 of hNGF with Ser residue present in mouse NGF. hNGFP61S “tagged” molecules are equally bioactive as hNGF [25] and are selectively detectable against wild type hNGF, with a specific monoclonal antibody [25].

The resulting double mutant hNGFP61S/R100E was expressed and purified from *E. coli* alongside hNGFR100E and hNGFP61S. The impact of the R100E mutation on binding affinities for the TrkA and p75NTR receptors is identical whether present in the context of human NGF or of the “tagged” hNGFP61S similarly reducing the binding affinity for p75NTR, while preserving the binding affinity for TrkA identical to that of hNGF or hNGFP61S [24]. In this study we assess the neurotrophic properties of hNGFP61S/R100E and its ability to prevent or revert neurodegeneration in different animal models.

### Reduced activation of PLC-1γ and Erk pathways by hNGFP61S/R100E

The activation of p75NTR and TrkA signal transduction pathways by hNGFP61S/R100E was studied in PC12 cells and primary hippocampal neurons, in comparison to hNGF, hNGFP61S and hNGFR100E. Beside the state of activation of TrkA, we decided to analyze downstream signaling pathways that are involved in NGF-mediated pain transmission and perception such as TrkA-activated Erk and PLC–1γ pathways and p75NTR-mediated c-jun activation [12], [26], [27].

**Figure 2:**
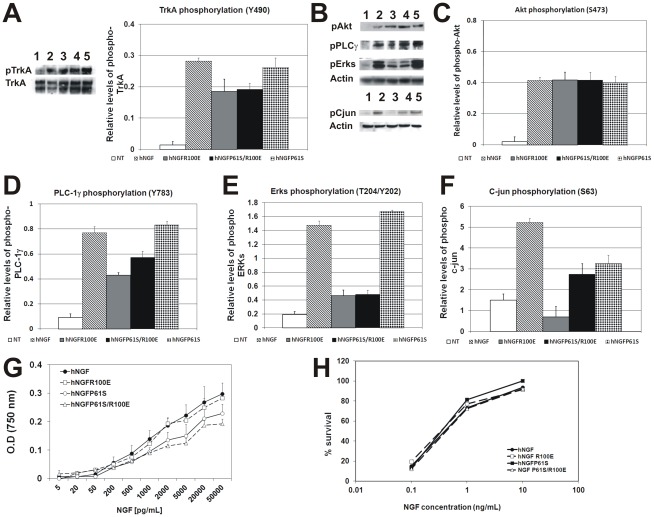
TrkA and p75NTR signalling by hNGFP61S/R100E. Densitometric analysis of Western blots for (**A**) TrkA (Y490), (**B**) Akt (S473), PLC-1γ (Y783), Erks (T204/Y202) and c-jun (S63) phosphorylation, in extracts from PC12 cells, stimulated for 30 minutes with 5 ng/ml of hNGF and hNGF mutants. Densitometric analysis of Western blots for (**C**) Akt, (**D**) PLC-1γ, (**E**) Erks and (**F**) c-jun. In all blots lanes 1 to 5 refer to untreated, hNGF-, hNGFR100E-, hNGFP61S/R100E- and hNGFP61S-treated cells, respectively. Values were normalized to total TrkA or actin, as described in the material and methods section. Graphs are correspond to three independent experiments. (**G**) Proliferation of human TF1 cells expressing TrkA by hNGF mutants. The proliferative index was equal to hNGF = 1.80; hNGFP61S = 1.68; hNGFR100E = 1.28 and hNGFP61S/R100E = 1.89 (**H**). The mutant hNGFP61S/R100E is as effective as hNGF, hNGFR100E and hNGFP61S in inducing survival of chick dorsal root ganglia, after 48 hours exposure. The experiments were performed in triplicate. Points and Bars represent the mean ± s.e.m.

The phosphorylation of TrkA residue Tyr490, which activates the Ras/MAP kinase cascade upon recruitment of ShC (reviewed in [28]), was largely unaffected by the R100E substitution, being only slightly reduced in PC12 cells treated with hNGFP61S/R100E, with respect to hNGF and hNGFP61S (Figure 2A). Likewise, hNGFP61S/R100E was equally effective as wild type hNGF, hNGFP61S and hNGFR100E in activating the Akt pathway (Figure 2B,C). The phosphorylation of residue Tyr 785 of TrkA by NGF leads to PLC-1γ recruitment and phosphorylation. A significant reduction of PLC-1γ phosphorylation was found after incubation of PC12 cells with hNGFP61S/R100E, with respect to hNGFP61S and wild type hNGF (Figure 2B,D), similarly to what observed for the hNGFR100E mutant (Figure 2B,D). The activation of Erk in PC12 cells was analyzed with antibodies against active Erk (residues Thr202/Tyr204). hNGFP61S/R100E, as well as hNGFR100E, significantly reduced Erk activation, with respect to hNGF and hNGFP61S (Figure 2B,E).

Binding of NGF to P75NTR triggers the phosphorylation of c-jun at residue Ser63 (reviewed in [28]. In hippocampal neurons, c-jun phosphorylation by hNGFP61S/R100E, as well as by hNGFR100E, determined with a site-specific anti- phospho c-jun antibody, was reduced by 30% with respect to that induced by hNGF or hNGFP61S (Figure 2F), consistently with the decreased binding affinity for p75NTR [24],

Thus, hNGFP61S/R100E shows an overall reduced p75NTR binding and signaling, and a subtle and selective impairment in the activation of some TrkA-dependent signalling pathways (notably PLC-1γ and ERK), while other signaling pathways are preserved (via Shc and Akt). The impact of the R100E mutation is therefore very similar, in the context of hNGF or of hNGFP61S [24].

### hNGFP61S/R100E promotes TrkA dependent cell proliferation as well as neuronal survival and differentiation

The ability of hNGFP61S/R100E to induce cell proliferation, survival and differentiation was tested in different cellular systems, in comparison to hNGF, hNGFP61S, hNGFR100E (globally named hNGF-X).

TrkA-dependent cell proliferation was evaluated in human erythroleukemia cells TF1, which respond in a dose-dependent manner to NGF [25], [29]. TF-1 cell proliferation provides a quantitative assay to determine the potency of hNGF mutants. The proliferation index for hNGFP61S/R100E was similar to that calculated for hNGF, hNGFP61S, hNGFR100E respectively, all comprised between 1 and 2 ng/ml (Figure 2G).

**Figure 3:**
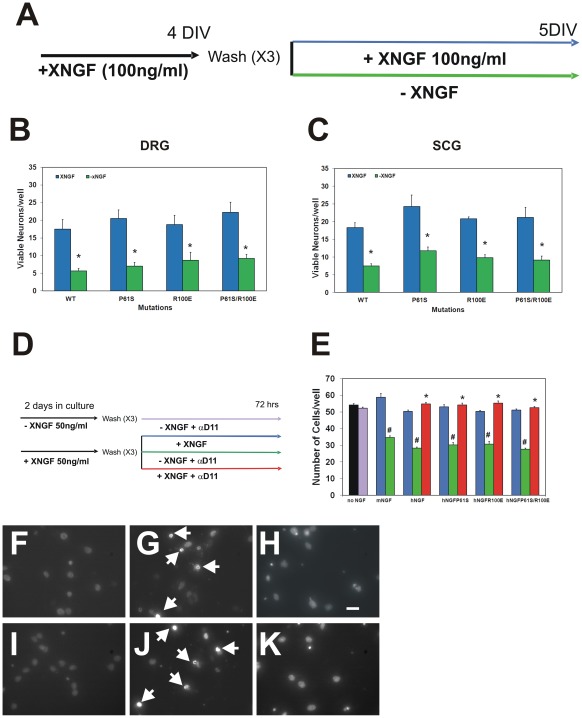
hNGFP61S/R100E bioactivity on sensory, sympathetic and hippocampal neurons. (**A**) Experimental scheme of NGF dependence in mouse dorsal root ganglia and superior cervical ganglia neurons and of hNGF induced survival. The mutant hNGFP61S/R100E is as effective as wild type hNGF in determining the survival and differentiation of mouse dorsal root ganglia sensory neurons (**B**) and mouse superior cervical ganglia (**C**), after a 4 days exposure. (**D**) Experimental scheme of induction of NGF dependence in rat hippocampal cells and hNGF induced survival. (**E**) Exposure of rat hippocampal cells to hNGF mutants (hNGFX), for 2 days in culture (priming) induces an NGF dependency (cell death after hNGF removal, green arm), which is rescued by re-exposing the cells to hNGFX (blue and red arms). The mutant hNGFP61S/R100E is equally effective in inducing dependency after priming and survival of rat hippocampal neurons (total cell counts after counterstaining with 4′,6-diamidino-2-phenylindole (DAPI). (**F–K**) Deprivation of hNGF (G) and of hNGFP61S/R100E (J) induces caspase −3 activation (arrows), compared to neurons exposed to the corresponding hNGF mutants (F,I). Exposure to hNGF (H) and hNGFP61S/R100E (K) overcomes the cell death induced by anti-NGF addition. Cells were counterstained with 4′,6-diamidino-2-phenylindole (DAPI) The field is the merge of the two separate channels used to acquire the caspase and the DAPI images. Scale bar in F–K = 50 µm.

Survival curves for chick embryonic DRG neurons [30] exposed to wild type hNGF or to hNGF mutants were totally superimposable (Figure 2H), as were those obtained when the activity of the hNGFP61S/R100E mutant was tested in cultures from mouse dorsal root ganglia (DRG) and superior cervical ganglia (SCG). Mouse DRG and SCG cultures were first exposed for 4 days to 100 ng/ml of hNGF or of each hNGF mutants (Figure 3A). On the fifth day, DRG and SCG neurons were deprived of the corresponding neurotrophin for 24 hours, before cell counting (Figure 3A) In mouse DRG cultures, the survival efficacy of hNGFP61S/R100E, was identical to that of hNGF, hNGFP61S and hNGFR100E (Figure 3B). Likewise, we found that in cultures of mouse SCG, the survival activity exerted by hNGFP61S/R100E was identical to that of hNGF, hNGFP61S and hNGFR100E (Figure 3C).

The neurotrophic and neuroprotective properties of hNGFP61S/R100E were then assessed in a neuronal NGF-dependent amyloidogenic model [8], based on hippocampal neuronal primary cultures. In this model, hippocampal neurons are first primed with NGF. Subsequent removal of NGF causes a γ–secretase dependent production of Aβ, that causes ensuing cell death [8]. In this system (Figure 3D), rat hippocampal neurons not “primed” with hNGF do not acquire NGF dependency (Figure 3E). On the contrary, when primed with NGF for 2 days, hippocampal neurons become dependent on NGF and die in the absence of NGF (Figure 3E). hNGFP61S/R100E was equally effective as hNGF, hNGFP61S and hNGFR100E in inducing priming and NGF dependency, as shown by the equivalent extent of neuronal death following its removal and neutralization with anti-NGF antibodies (Figure 3E, F–K). In sister cultures of hippocampal neurons, primed with each of the hNGF-X molecules, hNGF, hNGFP61S, hNGFR100E and hNGFP61S/R100E were equally effective in overcoming the cell death (Figure 3E, F–K). These results highlight the effectiveness of hNGFP61S/R100E in a disease-relevant neurodegeneration model linking neurotrophin deprivation to amyloidogenesis.

Finally, the pro-differentiative capacity of hNGFP61S/R100E was evaluated in rat PC12 and in human SH-SY5Y cells which are known to differentiate when incubated with NGF [31]. Priming and survival of rat PC12 cells induced by hNGFP61S/R100E was identical to that induced by hNGFR100E and by the positive controls hNGF, hNGFP61S (Figure S1A, B, C, D, E), both in terms of time course and of outcome. Notably, in the context of hNGFP61S the mutation R100E does not even induce the slight decrease in the number of processes/cell that was observed with hNGF/R100E, with respect to hNGF or hNGFP61S (Figure S1F). In human SH-SY5Y neuroblastoma cells [32], hNGFP61S/R100E was equally effective as hNGF, hNGFP61S, hNGFR100E in the neurite outgrowth induction (Figure 1G, H, I, J, K).

Thus, we conclude that the priming, survival, pro-differentiative and neuroprotective activity of hNGFP61S/R100E in different cell lines and neuronal cultures is identical to that of hNGF, hNGFP61S and hNGFR100E.

### Effect of hNGFP61S/R100E on pain induction

**Figure 4:**
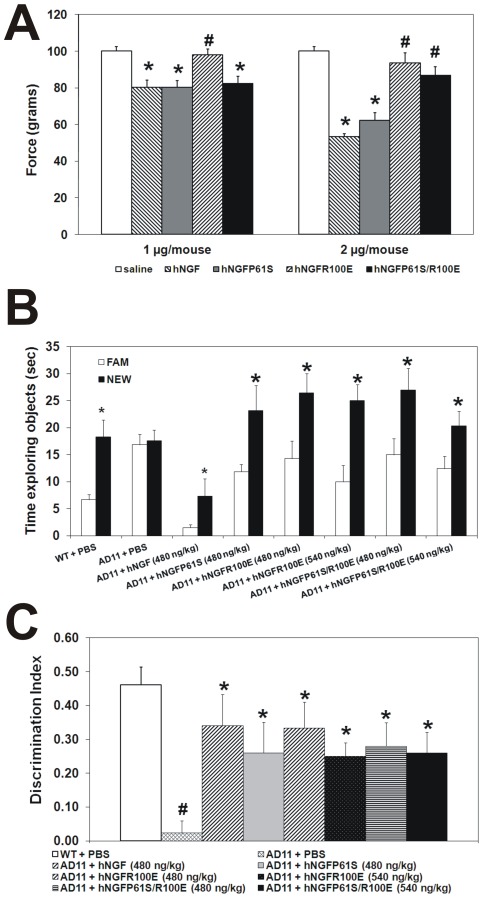
Reduced nociceptive response triggered by hNGFP61S/R100E and behavioural analysis of painless hNGF-treated AD11 mice. (**A**) Mechanical hyperalgesia: reduced hyperalgesic response 5 hours after intraplantar injection of 1 and 2 µg/mouse of hNGFR100E and hNGFP61S/R100E compared to hNGF. Bars represent mean ± s.e.m. ANOVA plus post-hoc Tukey-Kramer test; p<0.001. **B–C**, AD11 mice treated with hNGF or hNGFR100 mutants show a comparable, and complete, rescue of the memory impairment observed in saline-treated AD11 mice (**B**) During the test phase of ORT, WT mice and AD11 mice treated with different hNGF mutants explore more the new object than the familiar one. AD11 mice treated with PBS do not discriminate between familiar and new objects. (**C**) Discrimination index showing rescue of the memory deficit in AD11 mice treated with different hNGF mutants. Bars represent mean ± s.e.m, ANOVA plus post-hoc Holm-Sidak test, P<0.05.

NGF is involved in pain transmission and perception [12]. The nociceptive activity of NGF is mediated through the activation of TrkA and p75NTR receptors on sensory nerve endings and of the corresponding downstream signaling cascades, particularly TrkA-activated Erk and PLC–1γ pathways and p75NTR-mediated c-jun activation [12], [26], [27]. Since hNGFP61S/R100E binds p75NTR with a reduced affinity and shows a selective reduction of some of the TrkA-mediated signaling cascades involved in pain transmission (such as PLC-1γ), we verified whether this protein has a reduced nociceptive activity, using a paradigm of nociceptive sensitization by NGF [33]. Mechanical hyperalgesia was measured in adult CD–1 mice. Since robust interstrain variability was observed in nociceptive sensitivity, attention was paid the choice of the correct strain. We decided to use CD-1 mice since they provide a good nociceptive response, which is more stable than that observed in other mouse strains [34], [35].Mice were intraplantarly (i.pl.) injected with increasing doses of hNGFP61S/R100E. Besides saline injection, as a negative control, further controls were represented by the mutant hNGFP61S, which shows an overall bioactivity totally super imposable to that of wild type hNGF [25], and by hNGFR100E, which is characterized by a strong reduction of pro-nociceptive activity [21]. In the mechanical allodynia assay, hNGF determines a pain threshold dose response curve in the range of 0,5 µg to 4 µg per injection, with the dose of 4 µg inducing the maximum variation in pain threshold [21]. At each dose tested, the hyperalgesic effects are maximal 5 hours after the injection. In order to compare the efficacy of hNGFP61S/R100E to that of hNGFP61S and hNGFR100E, the sub-optimal dose-response range from 1 to 2 µg was explored, which is in the linear range, below the maximum saturation dose. Hyperalgesia was evaluated at the 5 hour time point. We observed the expected significant increase in hyperalgesia in hNGFP61S injected mice, at both doses (Figure 4) as well as the reported significant reduction by hNGFR100E [21] in elicited hyperalgesic effects (Figure 4), at both doses tested. hNGFP61S/R100E, at the dose of 2 µg/injection, showed a reduced hyperalgesic effect (Figure 4.), comparable to that seen for hNGFR100E, while, unlike the latter, it showed no difference from hNGFP61S at the lower dose of 1 µg/injection (Figure 4).

We conclude that hNGFP61S/R100E mutant displays a reduced effectiveness in nociceptor sensitization and in eliciting nociceptive responses in mice, with respect to hNGFP61S.

### Intranasal delivery of hNGFP61S/R100E prevents learning and memory deficits and neurodegeneration in aged anti-NGF AD11 and APPxPS1 mice

The properties demonstrated so far for hNGF R100 mutants in receptor binding and signaling assays, in different cellular models and in nociception sensitization in vivo assays [[21], [24] and results in this paper above], justify their prospective use as therapeutic candidates for neurodegenerative diseases. To this aim, the effectiveness of the hNGFR100 mutants in the prevention or rescue of neurodegeneration needs to be tested in adequate models *in vivo*. This was done in two different neurodegeneration mouse models, an NGF deprivation model (AD11 anti-NGF mice) and an Alzheimer FAD-based model (APPxPS1 mice), in which the cause of neurodegeneration is not linked to NGF deprivation.

The AD11 model displays progressive memory deficits and neurodegeneration as a consequence of NGF deprivation, induced by the expression of anti-NGF antibodies [36], [37]. The intranasal delivery of NGF was previously shown to prevent [18], [20] and rescue [18], [25] neurodegeneration in this model and thus it was used as a standard reference model to assess the biological activity of the hNGFR100E and hNGFP61S/R100E mutants *in vivo*. AD11 mice were treated with hNGF mutants at an age (6 months) when the progressive neurodegeneration is started, but not yet fully blown [18]. Two different concentrations of hNGF mutants (480 and 540 ng/kg, corresponding to 0.45 and 0.51 pmoles/injection) were chosen. The 0.45 pmole dose was found, in previous work on NGF delivery to AD11 mice, to be in the right part of the dose-response curve, corresponding to optimal pharmacological activity in this model [19], while the 0.51 pmoles dose was chosen on the basis of the IC50 for hNGFP61S/R100E in the TF-1 proliferation assay (see Figure 2G).

After two weeks of hNGF treatment, AD11 mice were tested for visual memory deficits in the object recognition test (ORT), the first behavioral deficit seen in the progression of AD11 neurodegeneration. Figure S2A describes the experimental validation of the behavioral assay, showing that all animal groups spend an equivalent time exploring the objects. AD11 mice treated with hNGF or the various hNGFR100 mutants showed a complete and comparable rescue of the memory impairment (Figure 4B,C), as shown by the longer time exploring the new object, relatively to the old familiar object.

**Figure 5:**
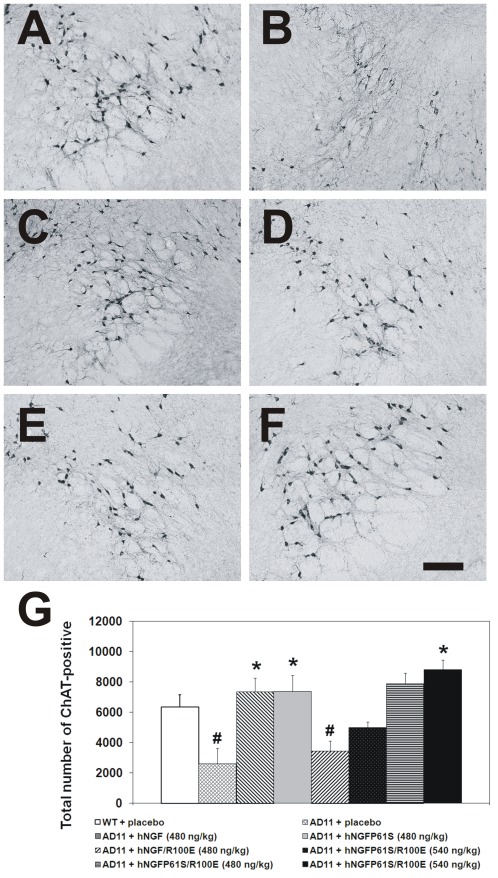
Effects of hNGFR100E and hNGFP61S/R100E in AD11 anti-NGF mice. Rescue of the neurodegenerative phenotype after intranasal delivery of hNGF mutants: Cholinergic deficit: (A) WT and (B) AD11 mice treated with PBS. AD11 mice treated, respectively with (C) hNGF (480 ng/kg); (D) hNGFP61S (480 ng/kg) (E) hNGFR100E (540 ng/kg) and (F) hNGFP61S/R100E (540 ng/kg) Scale bar = 200 µm. (**G**) Quantification of cholinergic neurons in the medial septum. Bars represent mean ± s.e.m, ANOVA plus post-hoc Holm-Sidak test, P<0.05.

**Figure 6:**
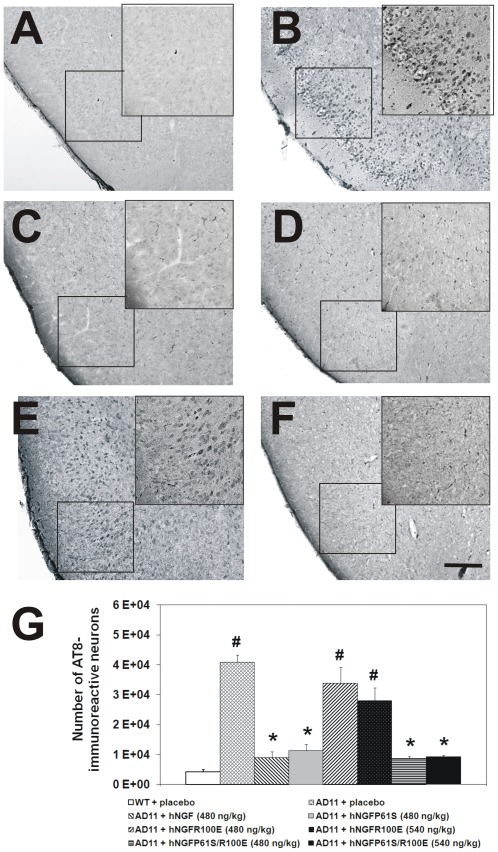
Rescue of the neurodegenerative phenotype after intranasal delivery of hNGF mutants in AD11 anti-NGF mice: Tau hyperphosphorylation. (A) WT and (B) AD11 mice treated with PBS. AD11 mice treated, respectively with (C) hNGF (480 ng/kg); (D) hNGFP61S (480 ng/kg) (E) hNGFR100E (540 ng/kg) and (F) hNGFP61S/R100E (540 ng/kg) Scale bar = 200 µm. (**G**) Quantification of AT8-positive neurons in the lateral entorhinal cortex. Bars represent mean ± s.e.m, ANOVA plus post-hoc Holm-Sidak test, P<0.05.

**Figure 7:**
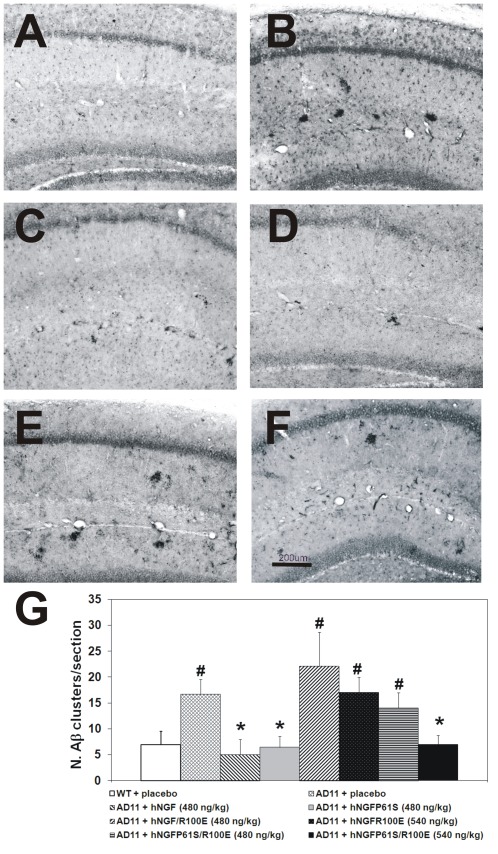
Rescue of the neurodegenerative phenotype after intranasal delivery of hNGF mutants in AD11 anti-NGF mice: β-amyloid. (A) WT and (B) AD11 mice treated with PBS. AD11 mice treated, respectively with (C) hNGF (480 ng/kg); (D) hNGFP61S (480 ng/kg) (E) hNGFR100E (540 ng/kg) and (F) hNGFP61S/R100E (540 ng/kg) Scale bar = 200 µm. (**G**) Quantification of Aβ-clusters in the hippocampus. Bars represent mean ± s.e.m, ANOVA plus post-hoc Holm-Sidak test, P<0.05.

After the behavioral assessment, mouse brains were evaluated at the neuropathological level by immunohistochemistry. Saline-treated AD11 mice displayed a marked reduction in the number of ChAT-positive neurons in basal forebrain nuclei (Figure 5B,G) and a typical increase in phosphorylated tau (Figure 6B,G) and clusters of Aβ-positive dystrophic neuritis (Figure 7B,G) with respect to non transgenic mice(Figure 5A, 6A and 7A), as described [18], [19].

AD11 mice treated with the higher dose of hNGFP61S/R100E showed a statistically significant increase in the number of ChAT-positive neurons (Figure 5F,G.), and a concomitant decrease in the number of phosphotau-positive neurons and of clusters of Aβ-positive dystrophic neurites (Figure 6F,G and Figure 7F,G, respectively). The efficacy of hNGFP61S/R100E was statistically indistinguishable from that of a similar dose of hNGF or hNGFP61S. On the other hand, the mutant hNGFR100E was less effective than hNGFP61S/R100E, determining only a 50% increase of the number of ChAT positive neurons at the higher dose (Figure 5E,G), but no significant effect on the number of phosphotau-positive neurons (Figure 6E,G) or of Aβ-positive dystrophic neurites (Figure 7E,G).

On the basis of these results, the hNGFP61S/R100E candidate was selected for further analysis, in a neurodegeneration model not dependent on NGF neutralization, the APPxPS1 mice (B6C3-Tg(APP695)3Dbo Tg(PSEN1)5Dbo/J) [38], based on FAD mutations in APP and PS1 genes (APP gene Swedish mutations K595N, M596Land presenilin 1 gene mutation A246E. APPxPS1 mice were treated intranasally with hNGFP61SR100E protein (540 ng/kg, 0,51 pmoles/injection), starting from 10 months of age for two months. At this age, the APPxPS1mice show no learning and memory deficits [39] and Aβ plaque deposition has only just started [38]. The design of the hNGFP61SR100E treatment is therefore addressing a potential preventive efficacy, in the early phases of the neurodegeneration.

**Figure 8:**
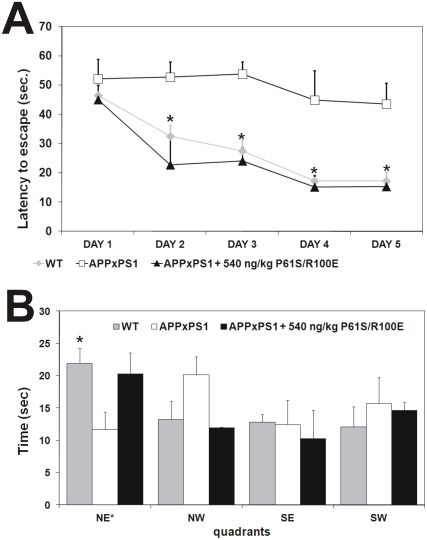
Effect of hNGFP61S/R100E intranasal administration to APPxPS1 mice: Spatial memory Morris water maze test. Spatial memory was determined by (**A**) latency and (**B**) probe test measures. P<0.01 in the learning phase by ANOVA followed by post-hoc Holm-Sidak analysis comparing APPxPS1 treated mice to APPXPS1 treated with saline.

**Figure 9:**
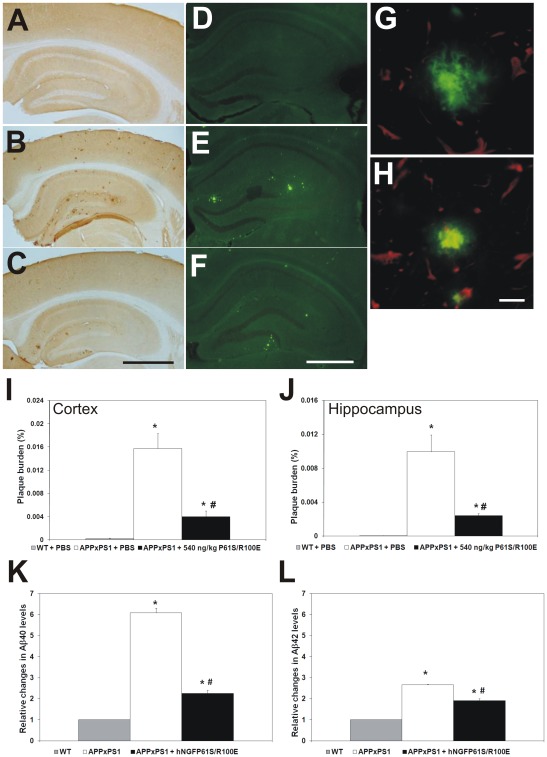
Effects of hNGFP61S/R100E on Aβ plaque load revealed by immunohistochemistry, Thioflavin S staining and ELISA. (**A,D**) Absence of Aβ plaques in the cortex and hippocampus of WT mice (**B,E**) Presence of Aβ plaques in the brain of APPxPS1. (**C,F**) The intranasal administration of 540 ng/kg of hNGFP61S/R100E decreases the plaque load. (**G,H**) The treatment with hNGFP61S/R100E determined a shift from fibrillar (**G**) to more compact morphology of the remaining plaques (**H**). (**I, J**) Stereological quantification of plaque load in the cortex and hippocampus. (**K,L**) Histograms showing the reduction in the relative levels of soluble (**K**) Aβ1–40 and (**L**) Aβ1–42 in APPxPS1 brains after treatment with hNGFP61S/R100E. Points and bars represent mean ± s.e.m. ANOVA followed by post-hoc Holm-Sidak analysis: P<0.05. Scale bars in A–F = 1000 µm. Scale bars in G–H = 50 µm.

After a two months treatment, spatial learning and memory was tested in the Morris water maze. First of all, the swimming speed and distance travelled during the first trial did not differ among the experimental groups (Figure S2B,C, respectively). PBS treated APPxPS1 mice showed a clear deficit in spatial learning (Figure 8A) and memory of the learned task (Figure 8B.). Both deficits were improved, to levels identical to control littermates, in hNGFP61S/R100E-treated APPxPS1 mice (Figure 8). The brains were subsequently analysed at the neuropathological level by immunohistochemistry. Aβ immunohistochemistry and Thioflavin S staining confirmed the presence of Aβ plaque deposits in the cortex and hippocampus of PBS treated APPxPS1 mice (Figure 9 B,E,I,L). No Aβ plaques were observed in WT transgenic mice (Figure 9A,D,I,J). Strikingly, hNGFP61S/R100E substantially reduced the plaque load in the hippocampus and cortex of APPxPS1 mice (Figure 9C,F, I,J) and determined a shift from a fibrillar to a more compact morphology of the residual plaques (Figure 9G,H). The reduction of Aβ in hNGFP61S/R100E treated APPxPS1 mice was confirmed at the biochemical level, by ELISA, showing a significant reduction of the soluble pools of both Aβ 1–40 and Aβ 1–42 with respect to the PBS-treated APPxPS1 mice (relative levels shown in Figure 9K,L).

A growing body of evidence shows that the most synaptotoxic form of the Aβ peptide is represented by oligomeric forms of the Aβ peptide.

**Figure 10:**
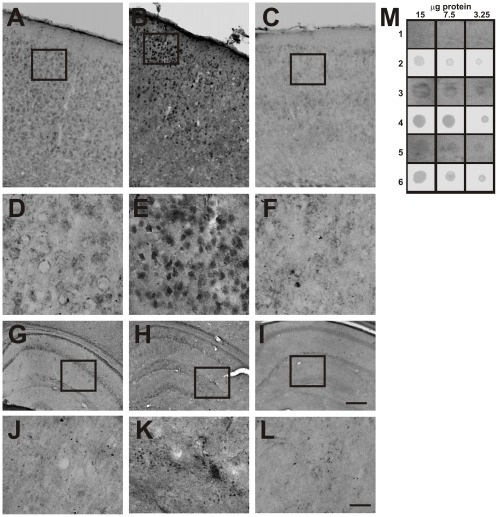
Decreased Aβ oligomer immunoreactivity in APPxPS1 mice after treatment with hNGFP61S/R100E: polyclonal pAb A11 antibody. (**A**) Parietal **c**ortex and (**G**) hippocampus of WT mice treated with PBS. Enlargements are shown in **D** and **J**, respectively. (**B**) Cortex and (**H**) hippocampus of APPxPS1 mice treated with PBS. Enlargements are shown in **E** and **K**, respectively. (**C**) Cortex and (**I**) hippocampus of APPxPS1 mice treated with PBS. Enlargements are shown in **F** and **L**, respectively. (**M**) Dot blot analysis of brains extracts from WT (row 1,2), APPxPS1 (rows 3,4) and APPxPS1 mice treated hNGFP61S/R100E (rows 5,6) probed with the A11 (row 1,3,5) and anti-tubulin (rows 2,4,6) antibodies. The image is representative of 4 independent experiments. Scale bars in A–B, G–I = 200 µm. Scale bars in D–F, J–L = 50 µm.

Since the anti-Aβ antibodies used for immunohistochemistry and ELISA do not distinguish between monomeric, oligomeric or fibrillar forms of this peptide, we examined the effect of the administration of hNGFP61S/R100E on the level of Aβ-oligomer. The commercial polyclonal anti-Aβ oligomers antiserum pA11 [40] and a recombinant antibody fragment selective for conformational oligomeric forms of Aβ, scFv A13 [41] were used. The conformational scFvA13 recombinant antibody binds selectively Aβ-oligomers (both mouse and human, and both ADDLs and natural Aβ-oligomers) and shows no binding activity for monomeric nor fibrillar Aβ [41]. The polyclonal pAb A11 antibody labels Aβ-oligomers in neurons of the cortex (Figure 10B,E) and hippocampus (Figure 10H,K) of APPxPS1 mice. On the contrary, no specific labeling was found in WT mice (Figure 10 A,D and G,J). Interestingly, the treatment with hNGFP61S/R100E significantly reduced the intracellular staining detected by the A11 antibody (Figure 10C, D, E, F, G, H, I, J, K, L).

Dot blot analysis of brain extracts from PBS-treated and hNGFP61S/R100E-treated APPXPS1 mice confirmed the efficacy of mutant hNGF in decreasing the levels of Aβ-oligomers in transgenic brains (Figure 10M).

**Figure 11:**
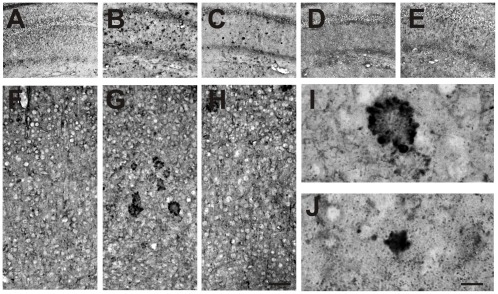
Decreased Aβ oligomer immunoreactivity in APPxPS1 mice after treatment with hNGFP61S/R100E: scFV A13 antibody. Hippocampus from (**A**) WT mice treated and (**B**) APPxPS1 mice treated with PBS and (**C**) APPxPS1 treated with hNGFP61S/R100E mice. In **D,E**, images from sections from APPxPS1 mice treated with PBS and used as experimental controls in which the primary antibody was omitted and sections were incubated only with the antibodies against the (**D**) SV5 tag and the biotinylated anti-rabbit antibody. In (**E**) the antibody against SV5 was also omitted from the incubation. In **F–H**, A13 labeled sections from the cortex of (**F**) WT mice treated and (**G**) APPxPS1 mice treated with PBS and (**H**) APPxPS1 treated with hNGFP61S/R100E mice. In **I** and **J**, Enlargements of Aβ oligomers immunoreactivity in (**I**) APPxPS1 and in (**J**) APPxPS1 mice treated with hNGFP61S/R100E. Scale bars in A–H = 100 µm; scale bar in I–J = 30 µm.

Similarly, we found that the scFvA13 antibody specifically labels Aβ-oligomers in the hippocampus (Figure 11B) and cortex (Figure 11G,I) of APPxPS1 mice, with no specific staining detected in WT mice (Figure 11A,F) nor in APPxPS1 sections in which the primary antibody fragment and/or the anti-V5 antibody were omitted (Figure 11D and E, respectively). Notably, the intranasal administration of hNGFP61S/R100E strongly reduces both the intracellular and extracellular staining for Aβ-oligomers (Figure 11 C,H,J), with respect to PBS-treated APPxPS1 mice (Figure 11B,G,I).

**Figure 12:**
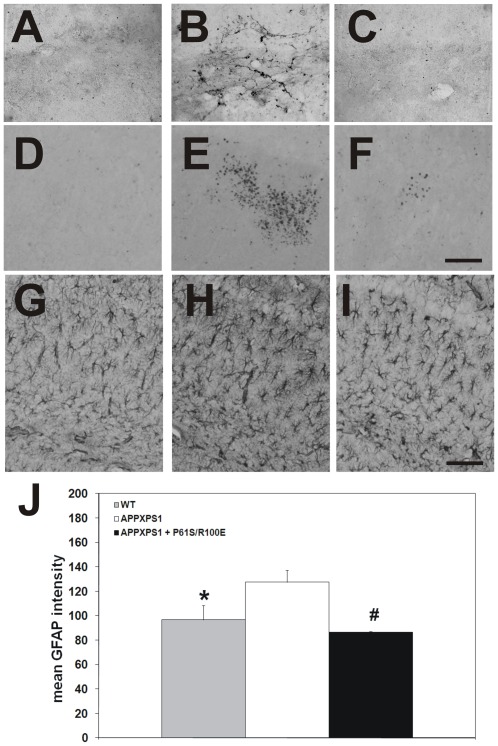
Reduced microgliosis and astrocytosis in APPxPS1 mice after intranasal hNGFP61S/R100E treatment. Absence of (**A,D**) microgliosis (revealed with anti-CD11b and anti-CD45 antibodies) and (**G**) astrocytosis (revealed with anti-GFAP antibodies) in the hippocampus of WT mice. Presence of (**B,E**) microgliosis and (**H**) astrocytosis in the brain of APPxPS1. The administration of hNGFP61S/R100E decreases (**C,F**) microgliosis and (**I**) astrocytosis. Images in A–C and D–F were obtained after incubation with the anti-CD11b and anti-CD45 antibodies, respectively. (**J**) Quantification of astrogliosis. Bars represent mean ± s.e.m. ANOVA followed by post-hoc Holm-Sidak analysis: P<0.05. Scale bars in A–F = 50 µm. Scale bars in G–I = 100 µm.

To start exploring the mechanism through which NGF reduces Aβ accumulation in APPxPS1 mice, we examined glial cells in the hippocampus. Indeed, in the hippocampus the majority of TrkA receptors, besides the TrkA receptors present on basal forebrain cholinergic terminals, are expressed by glial cells [42]. We investigated whether the treatment with hNGFP61S/R100E might modify the microgliosis (Figure 12B,H) and astrocytosis (Figure 12E; J present in APPxPS1 mice. hNGFP61S/R100E substantially reduced the number of CD11b (Figure 12C) and CD45 (Figure 12F) immunoreactive microglial cells and of GFAP-immunoreactive astrocytes (Figure 12I,J) ) in the hippocampus and cortex of APPxPS1 mice.

### Reduced pErks, c-jun and increased synaptophysin in APPXPS1 mice treated with hNGFP61S/R100E

Aβ oligomers have been shown to activate intracellular signaling pathways increasing the phosphorylation of Erks [43] and c-jun [44] and to lead to a reduction of synaptic proteins [45].

To further explore the mechanisms underlying the neuroprotective and anti-amyloidogenic effect of hNGFP61S/R100E, we evaluated by immunofluorescence, the levels of phosphoErks and phospho-c-jun in CA1 hippocampal neurons from brain sections from APPxPS1 mice treated with PBS and hNGFP61S/R100E.

**Figure 13:**
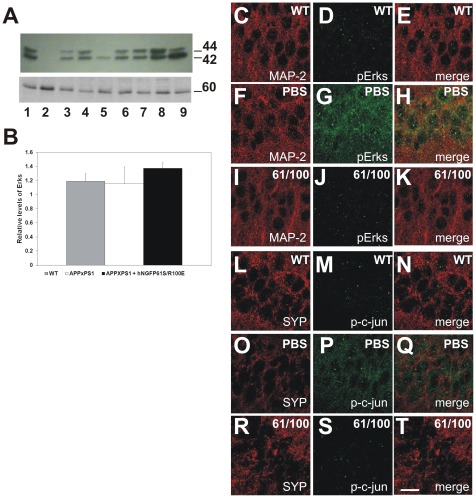
Reduced activation of phospho-Erks and p-c-jun and increased synaptophysin staining in APPxPS1 mice treated with hNGFP61S/R100E. (A) Western analysis of soluble brain extracts from WT, APPxPS1 and painless hNGF-treated APPxPS1 mice. Blots were probed with anti-Erks (upper panel) and mAb anti-tubulin YOL1 (lower panel). Lanes 1–3 refer to WT mice, lanes 4-6 to APPxPS1 mice treated with PBS and lanes 7–9 to APPxPS1 mice treated with hNGFP61S/R100E. (**B**) The graph reports quantitative determinations of the intensities of relevant bands. **C–K**, Immunofluorescence for MAP2 and pErks in WT mice (**C–E**), APPxPS1 mice treated with PBS (**F–H**) and (**I–K**) APPxPS1 mice treated with hNGFP61S/R100E (61/100). **L–T**, Immunofluorescence for synaptophysin (SYP) and pErks in WT mice (**L–N**), APPxPS1 mice treated with PBS (**O–Q**) and (**R–T**) APPxPS1 mice treated with hNGFP61S/R100E (61/100). Scale bar = 25 µm.

Western blot analysis of total Erks did not show any difference between the different groups of treatment (Fig. 13A,B).

We found that the levels of both phosphoErks (Figure 13F, G, H) and phospho-c-jun (Figure 13O, P, Q) are much higher in APPxPS1 mice treated with PBS than in WT mice (Figure 13C, D, E and L, M, N, respectively for phosphoErks and phospho-c-jun). Interestingly, in APPxPS1 mice treated with hNGFP61S/R100E the levels of both phosphoErks and phospho-c-jun were greatly reduced as in WT mice (Figure 13 I, J, K and R, S, T, respectively for phosphoErks and phospho-c-jun). Moreover, labeling with an anti-synaptophysin antibody showed that in APPxPS1 mice treated with PBS (Figure 13O) there is a decreased expression of this synaptic marker with respect to WT mice (Figure 13L), which is fully reverted by the intranasal administration of hNGFP61S/R100E (Figure 13R).

In conclusion, hNGFP61S/R100E appears to be effective in providing broad neuroprotection to APPxPS 1 double transgenic mice, thereby preventing the onset and progression of learning and memory deficits and reducing the amyloid load in this model. The neuroprotective effect by recombinant hNGF protein appears to be exerted at multiple possible levels, by slowing the generation of Aβ peptide and Aβ oligomers and by reducing the microgliosis and astrogliosis, possibly increasing the clearance of Aβ peptides, thereby leading to the observed reduced plaque load.

## Discussion

The well documented nociceptive actions of NGF represent a major drawback for the development of an NGF-based prospective therapy for human diseases [13], [46]. Thus, to develop NGF for AD therapy, invasive local delivery approaches are being currently adopted, involving the neurosurgical injection into the brain parenchyma of cells secreting NGF [14] or of viral particles harboring hNGF gene [15].

To fully exploit the therapeutic potential of NGF it is necessary to improve its therapeutic window, by increasing the access of NGF to CNS target regions, while limiting its off target, pain-inducing actions [7].

The intranasal delivery option provides a promising solution towards the former objective. Indeed, efficacy of intranasal NGF delivery [17] to rescue neurodegeneration in animal models has been demonstrated [18], [19], [20]. As to whether and how the pain inducing activities of NGF can be reduced or eliminated by this delivery route remained an open problem, since passage of NGF into the blood stream, from the nasal compartments, has been shown [16], [17]. NGF therapeutic window could be further increased if its nociceptive effects could be avoided altogether.

In this paper, we characterize a recombinant NGF variant that, while displaying a full neurotrophic and anti-amyloidogenic activity, also shows a reduced nociceptive activity. The hNGFP61S/R100E molecule combines a P61S tagging mutation [25], with the R100E mutation, designed to selectively reduce the pain sensitizing activity of NGF, while retaining its neurotrophic properties [21], [24]. On the hNGFP61S backbone, a second mutation was inserted at position R100, inspired by the genetic mutation found in NGFB gene in HSAN V patients, which changes the basic R100 in mature NGF to a non-polar tryptophan, and is responsible for the decreased perception of pain in these patients [22]. In recent studies we showed that the clinical phenotype of HSANV can be, at least in part explained by a selective inability of hNGF R100 protein to activate nociception, while retaining the neurotrophic properties [21]. Indeed, the binding affinity of hNGFR100 mutants to p75NTR is strongly reduced, while that for TrkA remains unchanged [21], [24]. Despite the identical TrkA binding affinity, hNGFR100 mutants differ from hNGF in their ability to activate downstream TrkA-dependent signalling pathways, with a notable selective reduction in the ability to activate PLC–1γ and Erks [21]. Notably, hNGFR100 mutants do not sensitize nociceptors and fail to induce pain *in vivo* [21]. Thus, hNGFR100 mutants may share some properties of other NGF mutants described to be selective TrkA agonists with respect to p75NTR [47], but have the additional property of selectively modulating a subset of the signaling pathways downstream of TrkA, and to be proven as less potent nociception activators.

On the basis of these properties, the hNGFP61S/R100E therapeutic candidate was designed, aimed at optimizing the therapeutic window for NGF. The characterization of the hNGFP61S/R100E double mutant is provided in this paper. Moreover, until recently [48] no study addressed the issue whether administration of NGF, or of NGF-related molecules, might affect the neurodegeneration in FAD related APP mouse models.

We show that hNGFP61S/R100E is characterized by a reduced ability to activate PLC–1γ, Erks and c-jun, providing a molecular explanation for the reduced pro-nociceptive activity, observed in a mechanical allodynia pain model. hNGFP61S/R100E showed no difference with respect the hNGF counterparts in a number of different survival, differentiation and proliferation cellular assays. Thus, hNGFR100P61S/R100E maintains the neurotrophic signaling stream unchanged, while showing a reduced signaling involved in nociceptor sensitization and pain perception. Notably, hNGFP61S/R100E is very effective to rescue or prevent neurodegeneration and learning and memory deficits in mouse models, including an APP-based model, in which the neurodegeneration is not directly caused by NGF inhibition.

We observed a higher effectiveness of hNGFP61S/R100E, with respect to hNGFR100E, in rescuing the neurodegeneration in the AD11 model, as well as a slight difference between the R100E and the P61S/R100E NGF proteins in nociceptive sensitization assays, most likely involving a structural cross-talk between S61 and R100 residues. Thus, the P61S substitution, which per se does not determine any measurable influence, compared to wild type NGF (Covaceuszach et al 2009), , could exert a long distance influence on the residue at position 100. In all the available 3D structures of hNGF complexes [49], [50], [51] loop 58–68 (CRDPNPVDSGC) is only partially resolved. Indeed the region 61–66 (PNPVDS), including the residue P61, is structurally not defined. On the contrary, in the crystal structures of mouse NGF (mNGF) [52], [53] the homologous loop (CRASNPVESGC), including S61, is structurally well defined. Thus, the 58–68 loop in hNGF has likely a higher intrinsic conformational flexibility compared to mNGF. This loop is structurally positioned over the C58–C108 and C68–C110 cysteine knot, and it may pose structural constraints on the possible conformations taken by the disulphide bridges of the cysteine knot. The C58–C108 and C68–C110 disulphide bridges pair β-strand-2 to β-strand-4. Residue R100 is located at the N-term of β-strand-4 (carrying C108 and C110). The P61S substitution in hNGF, by rendering the 58–68 loop structurally similar to that observed in mNGF, might cause a local conformational change in the proximity of the interacting cysteine knot, thereby exerting a long-distance effect on R100, which could explain the observed subtle modifications in the functional effects of this mutation in the context of hNGF or of hNGFP61S.

The neuroprotective and anti-amyloidogenic properties of hNGFP61S/R100E mutant demonstrated in APPxPS1 transgenic mice are noteworthy. In this mouse model, the expression of mutated human APP and PS1 is the first cause of neurodegeneration. The administration of hNGFP61S/R100E was performed at an age when memory deficits have not yet started [39] and amyloid plaque deposition is in the initial phase [38]. This allowed to study the effects of hNGFP61S/R100E at very early stages of the progressive neurodegeneration, showing that in APPxPS1 the hNGFP61S/R100E mutant was able to prevent learning and memory defects and to greatly reduce Aβ deposition. In addition, by using a conformation specific antibody anti-Aβ oligomers [41], we demonstrated that hNGFP61S/R100E prevents or reduces the accumulation of Aβ oligomers, considered the earliest and most synaptotoxic forms of Aβ. Thus, hNGFP61S/R100E exerts an anti-amyloidogenic effect *in vivo*, in a disease-relevant FAD-based model. From a mechanistic point of view, this might involve a generalized neuroprotective activity by the neurotrophin, at multiple levels, by slowing the generation of Aβ peptide and Aβ oligomers, by reducing the microgliosis and astrogliosis and/or, possibly, by increasing the clearance of Aβ peptides, thereby leading to the observed reduced plaque load and Aβ oligomer levels. The observed effects on astrocytes and microglia are consistent with both these cell types expressing TrkA and p75NTR NGF receptors, in normal and pathological conditions [54], [55], [56]. Reactive astrocytes, present in APP xPS1 brain due to amyloid pathology, also express higher levels of TrkA in human AD [57], [58]. We found a reduction of both microgliosis and astrocytosis in hNGFP61S/R100E treated APPxPS1 brains, suggesting that the ensuing reduced neuroinflammation might contribute to remove an environment permissive for the buildup of amyloid pathology and neurodegeneration. Further studies are required to verify whether the activation of TrkA signaling by hNGFP61S/R100E might regulate the known mechanisms of internalization and Aβ degradation by astrocytes [59], [60].

The selective modulation of TrkA and p75NTR downstream signaling pathways might also contribute to the neuroprotective action of hNGFP61S/R100E. Indeed, while the activation of the Akt signaling pathway, which is preserved by hNGFR100 mutants, is required for a neuroprotective action and is downregulated by soluble Aβ oligomers [61], Erks and c-jun activation have been linked to neurodegeneration and cell death. Oligomeric assemblies of Aβ have been found to up-regulate phospho-Erks [62], which, in turn, can lead to abnormal phosphorylation of tau, generation of dystrophic neurites and progressive neuronal degeneration [43], [63], [64]. Similarly, up-regulation of c-jun has been linked to cell death and tau phosphorylation in AD [65], and its down-regulation prevents the amyloidogenic cleavage of APP and the formation of amyloid plaques in AD mouse models [44]. Thus, the decreased activation of Erks and c-jun by hNGFP61S/R100E would uncouple their involvement in a positive feedback neurodegenerative loop and facilitate the down-regulation of the effects by the aberrant APP processing. Further studies are required at more later stages of neurodegeneration, when conflicting results about the “benefits” of Erks activation have been reported [66].

The possibility of hNGFP61S/R100E acting directly on APP processing and its direct consequences, such as the vicious cycle linking Aβ to the proNGF/NGF balance [67], [68] should also be considered for future investigation. In this respect, it should be noted that APPxPS1 mice show a defect in NGF retrograde axonal transport by cholinergic neurons, as a consequence of APP over-expression [69]. Even if this defect does not lead to overt cholinergic deficit, administration of NGF might provide a boost to cholinergic function which may contribute to limiting the negative consequences of the abnormal APP processing. In any event, our results unequivocally show that this hNGF variant can exert broad neuroprotection and prevent neurodegeneration originated by FAD linked mechanisms. Moreover, the results provide additional evidence for the tight links between the pathological processes observed in FAD-based mouse models and in anti-NGF mouse models [70], and add the evidence that TrkA agonists can improve learning and memory deficits in APP mice [48].

In conclusion, our results provide a strong rational basis for the development of “painless” NGF variant for therapeutic applications. The hNGFP61S/R100E property has the dual property of being traceable with respect to human NGF, *via* the substitution P61S [25], and of having a greatly reduced pain sensitization activity on sensory fibers, while displaying an identical neurotrophic activity as hNGF. These properties make hNGFP61S/R100E an ideal candidate for the development of a noninvasive therapy for AD, avoiding the need for localized delivery by direct injection of engineered cells or of viruses into the brain parenchyma. The reduced pain sensitizing actions will allow higher doses to be delivered through intranasal delivery, and reach the target areas in the CNS while keeping the peripheral concentrations of NGF below the threshold for nociception.

## Materials and Methods

### Transgenic mice

Six-months old C57BL×SSJL AD11 anti-NGF mice were produced by breeding single transgenic AD11-VH and AD11-VK mouse lines, as described [37]. Wild type (WT) mice were obtained by crossing non transgenic littermates of AD11 mice.

B6 transgenic mice expressing human PS1 gene, harboring the familial AD-linked A246E mutation and a chimeric mouse/human APP695gene, harboring a human Aβ domain with mutations (K595N and M596L) linked to Swedish familial AD pedigrees (APPswe), were purchased from Jackson Labs (Bar Harbour, ME). Non transgenic littermates were used as wild type (WT) control mice.

Mice were kept under a 12 hours dark to light cycle, with food and water *ad libitum*. Experiments were performed according to the national and international laws for laboratory animal welfare and experimentation (EEC council directive 86/609, OJ L 358, 12 December 1987).

### hNGF mutants expression and purification

hNGF mutant expression and purification were performed as previously described [24]. Briefly, hNGF mutants were expressed in *E. coli* as unprocessed proNGF, refolded in vitro from inclusion bodies, and mature NGF was obtained from in vitro-refolded proNGF, by controlled proteolysis, followed by chromatography [24].

### *In vitro* phosphorylation signaling assays

Rat PC12 pheochromocytoma cells [71] and primary cultures of hippocampal neurons [72] were used to assess NGF receptor activation and signaling, after incubation with hNGF or hNGF mutants. PC12 cells were cultured as described [21]. To evaluate the activation of signaling pathways, PC12 cells were incubated for 30 minutes in the presence of 5 ng/ml of the corresponding hNGF mutant. Hippocampal neurons were prepared from rat embryos (embryonic day 17–18 (E17–18)), from timed pregnant Wistar rats (Charles River Laboratories Italia, Calco, Italy). The hippocampus was dissected out in Hanks' balanced salt solution buffered with Hepes and dissociated *via* trypsin/EDTA treatment. Cells were plated at 1×10^6^ cells on 3.5-cm dishes pre-coated with poly-DL-lysine. After 2 days of culturing in Neurobasal medium with B-27 supplement and glutamax (Invitrogen), cytosine arabinofuranoside was added to reduce glial proliferation. Half of the medium was changed every 3–4 days. Neurons were incubated with hNGF or hNGF mutants (100 ng/ml) for 30 min 3–5 days after plating. PC12 cells and hippocampal neurons were lysed in cold RIPA buffer supplemented with phosphatase and protease inhibitors (50 mM Tris pH 7.4, 150 mM NaCl, 1% Triton X100, 1% Na deoxycholate, 10 mM EDTA, protease inhibitor cocktail (Roche Applied Science, Penzberg, Germany), 1 mM sodium orthovanadate, 50 mM NaF, 100 nM okadaic acid). Insoluble material was removed with a 5 min, 10,000 rpm centrifugation. The extracts were quantified using the Lowry test (Bio-Rad, Hercules, CA ) and normalized in the subsequent assays.

PC12 and hippocampal neuron extracts were processed for Western blot analysis as described before [21]. The following primary antibodies were used: anti-phosphotyrosine 490 TrkA antibody (Cell Signaling Technology, Danvers, MA; diluted 1∶1000), anti-phosphoserine 476 Akt antibody (Cell Signaling Technology, diluted 1∶1000), anti-phosphotyrosine 783 PLC-1γ antibody (Cell Signaling Technology, diluted 1∶1000), anti-phosphothreonine 202/phosphotyrosine 204 Erk1/2 antibody (Cell Signaling Technology, diluted 1∶1000), anti phosphoserine 63 c-jun antibody (Cell Signaling Technology, diluted 1∶500) and anti-TrkA antibody (R&D Systems Inc., Minneapolis, MN; diluted 1∶1000). The intensities of the immunoreactive bands were quantified and analyzed by using the National Institutes of Health (NIH) image analysis program ImageJ 1.44P after normalizing for protein content, evaluated by the intensity of total TrkA band in the case of phospho- TrkA or actin (Sigma-Aldrich, clone AC-40, diluted 1∶1000) in the case of the other signaling proteins.

### *In vitro* survival, proliferation and neurotrophic assays

#### TF1 cell proliferation assay

TF1 cells (ATCC-LGC Standards, Teddington, UK) assay was performed as described [21], [29]. Methodological details are provided in Methods S1.

#### PC12 cell and SH-S5SY differentiation assays

Culturing conditions and incubation protocol with hNGF mutant are described in Methods S2.

#### Hippocampal cell neurodegeneration assay

In this system, rat hippocampal neurons are rendered NGF-dependent by incubation with NGF for two days, after which, upon removal of NGF and addition of anti-NGF antibodies, cells activate aberrant amyloidogenesis that causes Aβ peptide-dependent cell death [8]. Hippocampal neurons were prepared as described above. Neurons were primed by exposing them to hNGF or hNGF mutants (50 ng/ml) for 48 h at 3–4 days after plating, washed 3 times with the medium and incubated for additional 48 h in presence of the anti-NGF monoclonal antibody αD11 [73] (10 µg/ml) or of anti-NGF plus the same mutant (50 ng/ml) used during the priming. Viable hippocampal neurons were quantified by counting the number of intact nuclei. The presence of cell death was revealed by active caspase-3 immunoreactivity, incubating cells with the anti-active caspase 3 antibody (1∶100, Cell Signaling Technology). Cells were counterstained with 4′,6-diamidino-2-phenylindole (DAPI).

#### DRG and SCG neurons survival assay

Chick embryonic dorsal root ganglia (DRG) (E6–E9) were collected, cleaned, trypsinized, dissociated and cultured in the absence or presence of hNGF mutants (0,1–10 ng/ml) as described [30]. Mouse superior cervical ganglia (SCG) and DRG were dissected from postnatal day 3 C57BlxSJL mice, enzymatically dissociated, and plated on 48 wells culture dishes. Neurons were maintained in DMEM supplemented with fetal bovine serum (10%), penicillin (1 U/ml), streptomycin (1 U/ml), and hNGF or hNGF mutants (100 ng/ml). After 4 days, a subset of neurons were washed and incubated in culture medium without the addition of neurotrophins for 48 hours. Neurons were lysed and cell counts performed.

### *In vivo* NGF-induced nociceptive assays

CD1 male mice, weighing 40–45 g, from Charles River Labs (Como, Italy) were used for nociceptive tests. Different groups of mice were used for mechanical allodynia behavioral testing. The pro-nociceptive inducing activity of hNGFP61S/R100E was compared to that of hNGFP61S and of hNGFR100E. Mice were intraplantarly (i.pl.) injected, on their hindpaws' plantar surface, with 20 µl of hNGF, hNGFP61S, hNGFR100E or hNGFP61S/R100E at concentrations corresponding to 1 and 2 µg/20 µl/mouse in saline 0.9% NaCl. From previous studies with hNGFR100E, these concentrations are in a linear dose-response range below the maximum saturation dose [21]. Control mice were injected with 20 µl of saline. Mechanical allodynia was measured 1 hour before (baseline) and 1, 3, 4 and 5 hours after i.pl. injections, as described [21].

All experiments were conducted according to national and international laws for laboratory animal welfare and experimentation (EEC Council directive 86/609, OJ L 358, 12 December 1987. Experimentation was approved by Italian Department of Health (approval n. 9/2006).

#### Intranasal delivery of hNGF mutants

In AD11 mice, the intranasal administration of hNGF mutants was performed on mildly anesthetized mice, as previously described [20], in a number of 8–10 mice per group. hNGF mutant doses of 480 ng/kg for and 540 ng/kg were used. The dose range was chosen to cover the potency range of R100E mutants, as determined in the quantitative TF1 proliferation cellular assay. hNGF was used only at the dose of 480 ng/kg. In APPxPS1 mice, the modality of administration was the same, except for the omission of the anesthesia, since preliminary data showed a reduced resistance of these mice to repeated intraperitoneal administrations of the anesthetic. It should be taken into consideration that anesthesia, in AD11 mice, is a practical precaution, to restrain the mice during the intranasal administration and optimize the delivery. The experimental protocol was approved by Italian Department of Health (approval n. 1/2011).

The mutants were diluted in 1 M phosphate-buffered saline (PBS, pH 7.4) at a quantity of 0.45 and 0.51 pmoles respectively for the doses of 480 ng/kg and 540 ng/kg, and were administered intranasally to mice, 3 µl at a time, alternating the nostrils, with a lapse of 2 min between each administration, for a total of 14 times. During these procedures, the nostrils were always kept open. As control treatments, AD11 and APPxPS1 mice were treated with PBS. The frequency of administration for intranasal delivery was three times per week (every 2 days). WT mice treated with PBS were used as a control group. Administrations were repeated for 7 times, over a 15 days period.

### Object recognition test

The visual object recognition test was performed as described before [19], Details are provided in Methods S3.

### Morris water maze

Morris water maze was used to establish whether aged APPxPS1 mice treated with the hNGFP61S/R100E showed an amelioration in spatial memory deficits. A circular water tank, made of aluminum (diameter, 120 cm; height, 40 cm) was filled to a depth of 25 cm with water (23°C ±), rendered opaque by the addition of a small amount of milk powder. Four positions around the edge of the tank were arbitrarily designated north (N), south (S), east (E), and west (W), which provided four alternative start positions and also defined the division of the tank into four quadrants: NE, SE, SW, and NW. A circular clear Perspex escape platform (diameter, 10 cm; height, 2 cm) was submerged 0.5 cm below the water surface and placed at the midpoint of one of the NE quadrant. Mice were trained with four trials per day for 4 days (with an inter trial interval of 30 min). The start position (N, S, E, or W) was pseudo-randomized across trials. Mice were allowed up to 60 sec to locate the escape platform, and their escape latency was recorded. On the fifth day, the fourth trial was substituted by a probe trial, during which the escape platform was removed from the tank, and the swimming path of each mouse was video-recorded over 60 sec, while it searched for the missing platform.

#### Determination of soluble Aβ1–40 and Aβ1–42

The analysis was performed on soluble brain extracts using the Brain ELISA test kit (Millipore). Tissues were processed according to the manufacturer instructions. The values for APPxPS1 mice and APPxPS1 mice treated with hNGFP61S/R100E were normalized against the corresponding values obtained from WT brain extracts.

#### Western blot analysis

Brain extracts were analyzed by SDS/PAGE (10% polyacrilamide gels) and western blot (WB). Nitrocellulose membrane were probed with the antibodies against Erks 42/44 (Santa Cruz) and, after stripping, with the anti-tubulin monoclonal antibody YOL-1 (kindly provided by Cesar Milstein, MRC Laboratory of Molecular Biology, Cambridge, UK). The intensities of the immunoreactive bands were quantified and analyzed by using the National Institutes of Health (NIH) image analysis software (NIH IMAGEJ) after normalizing for protein content, evaluated by the intensity of the tubulin band with mAb YOL-1.

Dot blot analysis was performed by spotting on nitrocellulose membrane increasing amounts of soluble extracts corresponding to 3.5, 7 and 15 µg of protein. Incubation was performed with pAb A11 (Millipore) diluted 1∶1000 in 5% skimmed milk/PBS plus 0.1% Tween 20.

### Histological and neurostereological analysis

After behavioral analysis, mice were anesthetized with an excess of 2,2,2-tribromethanol (400 mg/kg) and intracardially perfused with a 4% solution of paraformaldehyde in PBS. Brains were processed for immunohistochemical analysis as described before [36], [74]. The following primary antibodies were used: goat anti-choline acetyltransferase (ChAT;1∶500, Millipore, Billerica, MA); goat anti-NH2 terminus of Aβ (1.100; Santa Cruz, Santa Cruz, CA); mouse anti-β amyloid clone 6E10 (1∶250; Sigma, St Louis, MO, for APPxPS1 mice only) and mouse anti-human phosphotau recognizing Ser199 (1∶10 clone AT8; Pierce Endogen, Rockford, IL); mouse anti-GFAP (1∶1000 clone G-A-5; Sigma); goat anti-CD45 and CD11b (1.100; Santa Cruz, Santa Cruz, CA); anti-phosphothreonine 202/phosphotyrosine 204 Erk1/2 antibody (1∶100, Cell Signaling Technology), anti phosphoserine 63 c-jun antibody (1∶100, Cell Signaling Technology); anti-microtubule-associated protein 2a+b (1∶100, Sigma); anti-synaptophysin (1∶100, Santa Cruz). For double immunofluorescence, chicken AlexaFluor 488anti-rabbit antibodies and donkey AlexaFluor555 anti-mouse antibodies were used. Brains from APPxPS1 mice were also incubated with the rabbit polyclonal antibody directed against the C-terminus of β amyloid 1–42 (1∶500, Millipore, Billerica, MA). The Thioflavin S assay was performed by incubating section with a 0,5% Thioflavin S solution.

The detection of Aβ oligomers in brain sections was performed using the anti-Aβ oligomer polyclonal antiserum pAb A11 and the sequence and conformational specific antibody scFv A13 [41]. Incubation with the polyclonal antibody A11 was carried out at a 1∶1000 dilution. When scFv A13 was used to reveal Aβ oligomers, sections were incubated with 0.1 µg/ml of the scFv A13 antibody, followed by the anti-V5 antibody (Sigma, 1∶500) against the c-terminal tag on scFv A13 and a biotinylated anti-rabbit antibody (Vector) in presence of 30% Fetal bovine serum (FBS). At the end of the immunohistochemical procedures, neurostereological analysis was performed as described before [19].

Astrogliosis was evaluated by measuring the intensity of GFAP staining in the hippocampus, after keeping fixed the condition of microscope illumination, with the anti-GFAP antibody clone G-A-5.

### Statistical Analyses

Statistical analyses were performed using the Sigmastat v. 3.11 program (Systat Software, San Jose, CA). The alpha was set at 0.05 and a normality and equal variance test were first performed.

All values of behavioral tests to assess nociception are expressed as mean ± s.e.m of 8–10 animals per group. Two-way ANOVAs for repeated measures were used to analyze the effects of pharmacological treatments. Post-hoc comparisons were carried out using Tukey-Kramer test. Differences were considered significant at p<0.05.

## Supporting Information

Figure S1 **Survival and differentiation of PC12 and SH-SY5Y cells by hNGFP61S/R100E.** In panels **A–E**, PC12 cells were primed with 50 ng/ml of hNGF (B), hNGFP61S (C), hNGFR100E (D) or hNGFP61S/R100E (E) for 1 week and replated for 2 days in presence of 10 ng/ml of either hNGF or the respective mutant. Negative controls (A) are represented by cells incubated in absence of hNGF or hNGF mutants. (F) Quantification of the number of PC12 processes after exposure to the different neurotrophins. (G) Untreated human neuroblastoma SH-SY5Y cells are induced to differentiate when treated for 7 days with 100 ng/ml of hNGF (H), hNGFP61S (I), hNGFR100E (J) or with hNGFP61S/R100E (K). Bars represent the mean ± s.e.m. *, P<0,05 versus hNGF.
(TIF)

Figure S2 **Effect of hNGFP61S/R100E intranasal administration to AD11 and APPxPS1 mice: validation controls for behavioral experiments.** (**A**) WT, **AD11 mice treated with PBS and** AD11 mice treated with hNGF or hNGFR100 mutants show comparable exploration times during the sample phase of the ORT. (**B, C**) Morris water maze test in APPxPS1 mice. Mice from all genotypes show an equal performance in terms of (**A**) swimming speed and (**B**) distance covered during the first trial on the first day
(TIF)

Methods S1 **TF1 cell proliferation assay.**
(DOCX)

Methods S2 **PC12 cell and SH-S5SY differentiation assays.**
(DOCX)

Methods S3 **Object recognition test.**
(DOCX)
