# Correction: Intranasal “painless” Human Nerve Growth Factors Slows Amyloid Neurodegeneration and Prevents Memory Deficits in App X PS1 Mice

**DOI:** 10.1371/annotation/97b6c799-1ebc-4e7b-8f86-47c1130dc00e

**Published:** 2012-08-27

**Authors:** Simona Capsoni, Sara Marinelli, Marcello Ceci, Domenico Vignone, Gianluca Amato, Francesca Malerba, Francesca Paoletti, Giovanni Meli, Alessandro Viegi, Flaminia Pavone, Antonino Cattaneo

The word "Factor" is misspelled in the article title. The correct title is: Intranasal "painless" Human Nerve Growth Factor Slows Amyloid Neurodegeneration and Prevents Memory Deficits in App X PS1 Mice. The correct citation is: Capsoni S, Marinelli S, Ceci M, Vignone D, Amato G, et al. (2012) Intranasal “painless” Human Nerve Growth Factor Slows Amyloid Neurodegeneration and Prevents Memory Deficits in App X PS1 Mice. PLoS ONE 7(5): e37555. doi:10.1371/journal.pone.0037555 

