# Supplementary figures and images for: Intranasal “painless” Human Nerve Growth Factors Slows Amyloid Neurodegeneration and Prevents Memory Deficits in App X PS1 Mice
Source: PLoS One. 2012 May 30;7(5):e37555. doi: 10.1371/journal.pone.0037555 (PMC3364340; doi:10.1371/journal.pone.0037555)

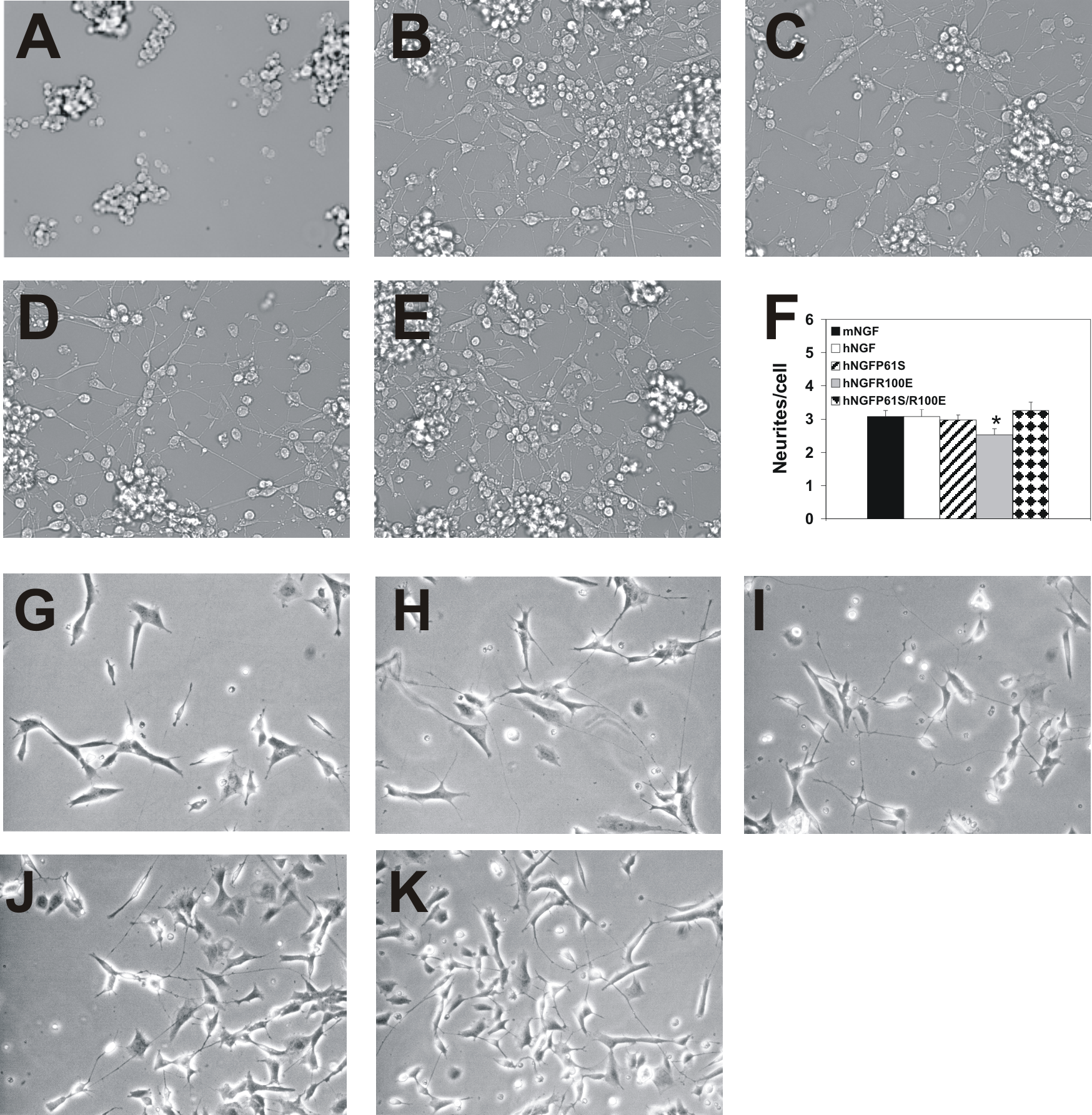

Supplement: Figure S1 — Survival and differentiation of PC12 and SH-SY5Y cells by hNGFP61S/R100E. In panels A–E, PC12 cells were primed with 50 ng/ml of hNGF (B), hNGFP61S (C), hNGFR100E (D) or hNGFP61S/R100E (E) for 1 week and replated for 2 days in presence of 10 ng/ml of either hNGF or the respective mutant. Negative controls (A) are represented by cells incubated in absence of hNGF or hNGF mutants. (F) Quantification of the number of PC12 processes after exposure to the different neurotrophins. (G) Untreated human neuroblastoma SH-SY5Y cells are induced to differentiate when treated for 7 days with 100 ng/ml of hNGF (H), hNGFP61S (I), hNGFR100E (J) or with hNGFP61S/R100E (K). Bars represent the mean ± s.e.m. *, P<0,05 versus hNGF. (TIF) [file pone.0037555.s001.tif]

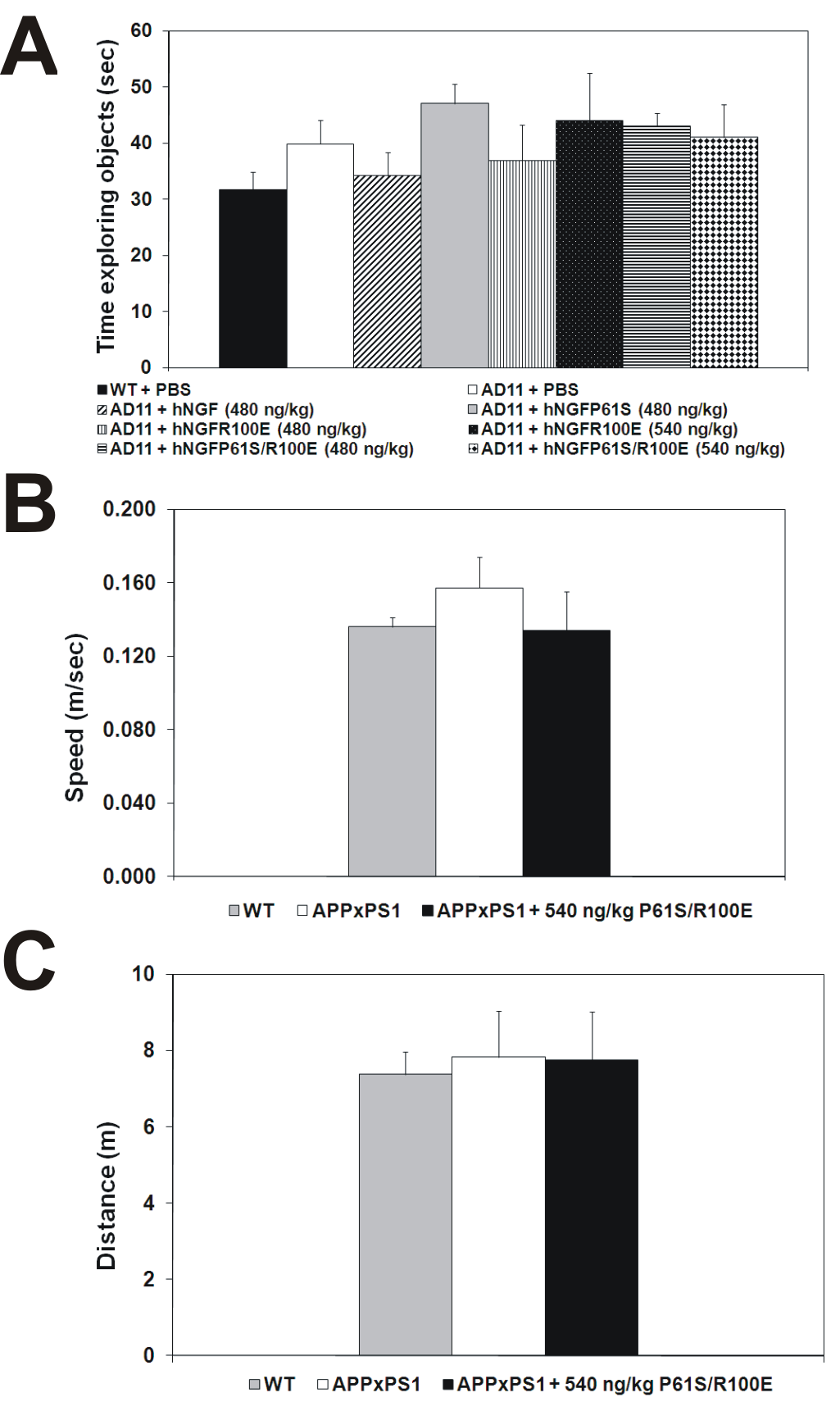

Supplement: Figure S2 — Effect of hNGFP61S/R100E intranasal administration to AD11 and APPxPS1 mice: validation controls for behavioral experiments. (A) WT, AD11 mice treated with PBS and AD11 mice treated with hNGF or hNGFR100 mutants show comparable exploration times during the sample phase of the ORT. (B, C) Morris water maze test in APPxPS1 mice. Mice from all genotypes show an equal performance in terms of (A) swimming speed and (B) distance covered during the first trial on the first day (TIF) [file pone.0037555.s002.tif]
