# Supplementary material for: Intranasal “painless” Human Nerve Growth Factors Slows Amyloid Neurodegeneration and Prevents Memory Deficits in App X PS1 Mice
Source: PLoS One. 2012 May 30;7(5):e37555. doi: 10.1371/journal.pone.0037555 (PMC3364340; doi:10.1371/journal.pone.0037555)
Supplement: Methods S1 — TF1 cell proliferation assay. (DOCX) [file pone.0037555.s003.docx]

***Methods S1. TF1 cell proliferation assay*.** TF1 cells (ATCC-LGC Standards, Teddington, UK) assay was performed as described [1]. TF1 were cultured for 1 week in RPMI 1640 medium with 2 mM L-glutamine adjusted to contain 1.5 g/l sodium bicarbonate, 4.5 g/l glucose, 10 mM HEPES, and 1 mM sodium pyruvate (90 %), supplemented with 2 ng/ml recombinant human GM-CSF (R&D Systems Inc.), and 10% fetal bovine serum. The TF1 proliferation assay was performed in 96 wells microtiter plates by incubating 15,000 cells per well in the presence of increasing doses of either wild type hNGF or the corresponding hNGF R100 mutants, ranging between 5 and 50,000 pg/ml. Cells were seeded 1h before adding treatments. A MTT cell proliferation assay (ATCC kit) was employed to evaluate the proliferative response: after a 40-hour culture period, MTT solution was added for an additional 4 hours incubation, according to the previously described colorimetric assay [2]. The intensity of each colorimetric signal was measured at 570 nm in a microtiter plate reader, 16 hours (overnight) following addition of detergent reagent. In all experiments, each treatment was done in triplicate.

1. Chevalier S, Praloran V, Smith C, MacGrogan D, Ip NY, et al. (1994) Expression and functionality of the trkA proto-oncogene product/NGF receptor in undifferentiated hematopoietic cells. Blood 83: 1479-1485.
2. Mosmann T (1983) Rapid colorimetric assay for cellular growth and survival: application to proliferation and cytotoxicity assays. J Immunol Methods 65: 55-63.
