# Supplementary material for: Intranasal “painless” Human Nerve Growth Factors Slows Amyloid Neurodegeneration and Prevents Memory Deficits in App X PS1 Mice
Source: PLoS One. 2012 May 30;7(5):e37555. doi: 10.1371/journal.pone.0037555 (PMC3364340; doi:10.1371/journal.pone.0037555)
Supplement: Methods S2 — PC12 cell and SH-S5SY differentiation assays. (DOCX) [file pone.0037555.s004.docx]

***Methods S2. PC12 cell and SH-S5SY differentiation assays*.** PC12 cells were maintained in RPMI 1640 medium (Invitrogen), supplemented with 5% fetal calf serum and 10% heat-inactivated horse serum, in presence of 100 ng/ml of hNGF or hNGF mutants. Alternatively, PC12 cells were primed with hNGF or hNGF mutants (50 or 100 ng/ml of NGF for 1 week) and then replated in the presence or absence of 10-50 ng/ml hNGF or hNGF mutants as described [1].

Human neuroblastoma SH-SY5Y cells (ATCC) were grown in DMEM/F12 Glutamax medium supplemented with 10% heat-inactivated fetal bovine serum, 2 mM glutamine, and 100 pg/ml gentamicin. For differentiation, cells were plated (1.5 x 10^4^ to 5 x 10^5^ cells/dish) in 35-mm Falcon dishes. After 5 days of 0.3 µM aphidicolin treatment, cells were allowed to differentiate for 7 days in presence of 100 ng/ml of hNGF or hNGF mutants [2] .

1. Cattaneo A, Capsoni S, Margotti E, Righi M, Kontsekova E, et al. (1999) Functional blockade of tyrosine kinase A in the rat basal forebrain by a novel antagonistic anti-receptor monoclonal antibody. J Neurosci 19: 9687-9697
2. LoPresti P, Poluha W, Poluha DK, Drinkwater E, Ross AH (1992) Neuronal differentiation triggered by blocking cell proliferation. Cell Growth Differ 3: 627-635.
