# Supplementary material for: Intranasal “painless” Human Nerve Growth Factors Slows Amyloid Neurodegeneration and Prevents Memory Deficits in App X PS1 Mice
Source: PLoS One. 2012 May 30;7(5):e37555. doi: 10.1371/journal.pone.0037555 (PMC3364340; doi:10.1371/journal.pone.0037555)
Supplement: Methods S3 — Object recognition test. (DOCX) [file pone.0037555.s005.docx]

***Methods S3. Object recognition test*** The apparatus consisted of a square arena (60 cm x 60 cm x 30 cm) constructed in PVC with black walls and white floor. The box and objects were cleaned up between trials to stop the build-up of olfactory cues. During the first day, mice received 2 habituation trials of 5 min duration in the empty box. The second session was delayed 30 min, after the end of the first one. The next day, each mouse was first placed in the box and exposed to two identical sample objects for 10 min (sample phase). The experimenter measured the total time spent in exploring the two objects. After a delay of 24 hours, the mice were placed back in the box and exposed to the familiar object and to a novel object, for a further 10 min (Test phase). The objects were placed in the same locations as the previous ones. The experimenter measured again the total time spent exploring each of the two objects. A discrimination index (DI) was calculated as the difference between the time spent exploring the new and the old object, divided by the total time spent exploring the objects. All trials were recorded using a videocamera connected to Any-maze^TM^ Video Tracking System v 4.5 (Stoelting Co., Wood Dale, IL).
